# A universal tool for marine metazoan species identification: towards best practices in proteomic fingerprinting

**DOI:** 10.1038/s41598-024-51235-z

**Published:** 2024-01-13

**Authors:** Sven Rossel, Janna Peters, Nele Charzinski, Angelina Eichsteller, Silke Laakmann, Hermann Neumann, Pedro Martínez Arbizu

**Affiliations:** 1 Senckenberg am Meer, German Centre for Marine Biodiversity Research (DZMB), 26382 Wilhelmshaven, Germany; 2 German Centre for Marine Biodiversity Research (DZMB), Senckenberg am Meer, 20146 Hamburg, Germany; 3 Marine Biodiversity Research, Institute of Biology and Environmental Sciences, Carl von Ossietzky University Oldenburg, 26129 Oldenburg, Germany; 4 Helmholtz Institute for Functional Marine Biodiversity at the University of Oldenburg (HIFMB), 26129 Oldenburg, Germany; 5 Alfred-Wegener-Institut Helmholtz-Zentrum für Polar- und Meeresforschung, Am Handelshafen 12, 27570 Bremerhaven, Germany; 6 Institute for Sea Fisheries, Thuenen Institute, 27572 Bremerhaven, Germany

**Keywords:** Biodiversity, Molecular ecology, Bioinformatics, Mass spectrometry, Proteomic analysis, Biological techniques, Ecology, Molecular biology, Ocean sciences

## Abstract

Proteomic fingerprinting using MALDI-TOF mass spectrometry is a well-established tool for identifying microorganisms and has shown promising results for identification of animal species, particularly disease vectors and marine organisms. And thus can be a vital tool for biodiversity assessments in ecological studies. However, few studies have tested species identification across different orders and classes. In this study, we collected data from 1246 specimens and 198 species to test species identification in a diverse dataset. We also evaluated different specimen preparation and data processing approaches for machine learning and developed a workflow to optimize classification using random forest. Our results showed high success rates of over 90%, but we also found that the size of the reference library affects classification error. Additionally, we demonstrated the ability of the method to differentiate marine cryptic-species complexes and to distinguish sexes within species.

## Introduction

Correct and cost-effective species identification is crucial in various research areas, including biodiversity assessments, where obtaining reliable information on species’ occurrences and distributions is pivotal. If species cannot be morphologically identified to the species level, they are often assigned to higher taxonomic levels, leading to less detailed analyses and consequently imprecise conclusions^1,2^. However, identification of samples using COI-barcoding is expensive, time consuming^3^ and therefore not feasible in large biodiversity assessments including large numbers of specimens.

Matrix Assisted Laser Desorption/Ionization Time-of-flight mass spectrometry (MALDI-TOF MS) is a rapid species identification method that measures a proteome fingerprint to identify specimens using a reference library. With few preparation steps, peptides and proteins are extracted from tissue and embedded in a matrix absorbing laser radiation while measuring ionized, intact compounds in a mass spectrometer^4^. This method is routinely applied for the identification of microorganisms such as bacteria, viruses and fungi^5–7^. It was also used for food fraud detection^8,9^ or to check food adulteration^10^. In pilot studies, it was successfully applied for identification of metazoans such as copepods^11–17^, isopods^18,19^, different groups of Cnidaria^20–22^, molluscs^23^, fish^8,24^ and especially disease vectors such as ticks, sandflies or mosquitoes^25–29^. Most studies only analyzed a few species or were limited to a certain taxonomic group while studies across different classes and phyla are still missing. Also, no gold standard protocol for metazoan analytics has been established yet. Systematic tests, how data processing will affect the identification success and whether and how pipelines need to be adapted to higher-taxonomic-level identification are also missing.

For the first time, we present a generalized workflow for species identification of metazoans as well as the subsequent bioinformatics using a wide spectrum of marine taxa. We emphasize the importance of adjusting bioinformatics to the data set and finally prove the power of proteomic fingerprinting for differentiation of morphologically cryptic, closely related marine species and beyond mere species identification on sex level, making it a promising tool for ecological research.

To investigate this, we start by looking at the sample preparation in terms of tissue to matrix ratio and how this effects mass spectra quality. This is followed by identifying the crucial steps during data processing for classification using random forest (RF). Subsequently, we analyze how large reference libraries should be to optimize rf-model capabilities for identification. Finally, we apply these findings to our dataset of almost 200 marine taxa to test both species identification as well as classification on a higher taxonomic level.

## Results

The data set contained 1246 specimens from 198 taxa including echinoderms (Asteroidea, Echinoidea and Ophiuroidea), molluscs (Bivalvia, Gastropoda, Polyplacophora and Cephalopoda), arthropods (Crustacea, Pantopoda) and chordates (Tunicata, Vertebrata: Teleostei, Elasmobranchii). For 1139 specimens a published COI barcode or another molecular identifier is available (Supplementary Table S1). The remaining specimens were identified morphologically. For 226 specimens attempts to obtain mass spectra either failed or were of minor quality and discarded.

### Sample preparation

**Figure 1:**
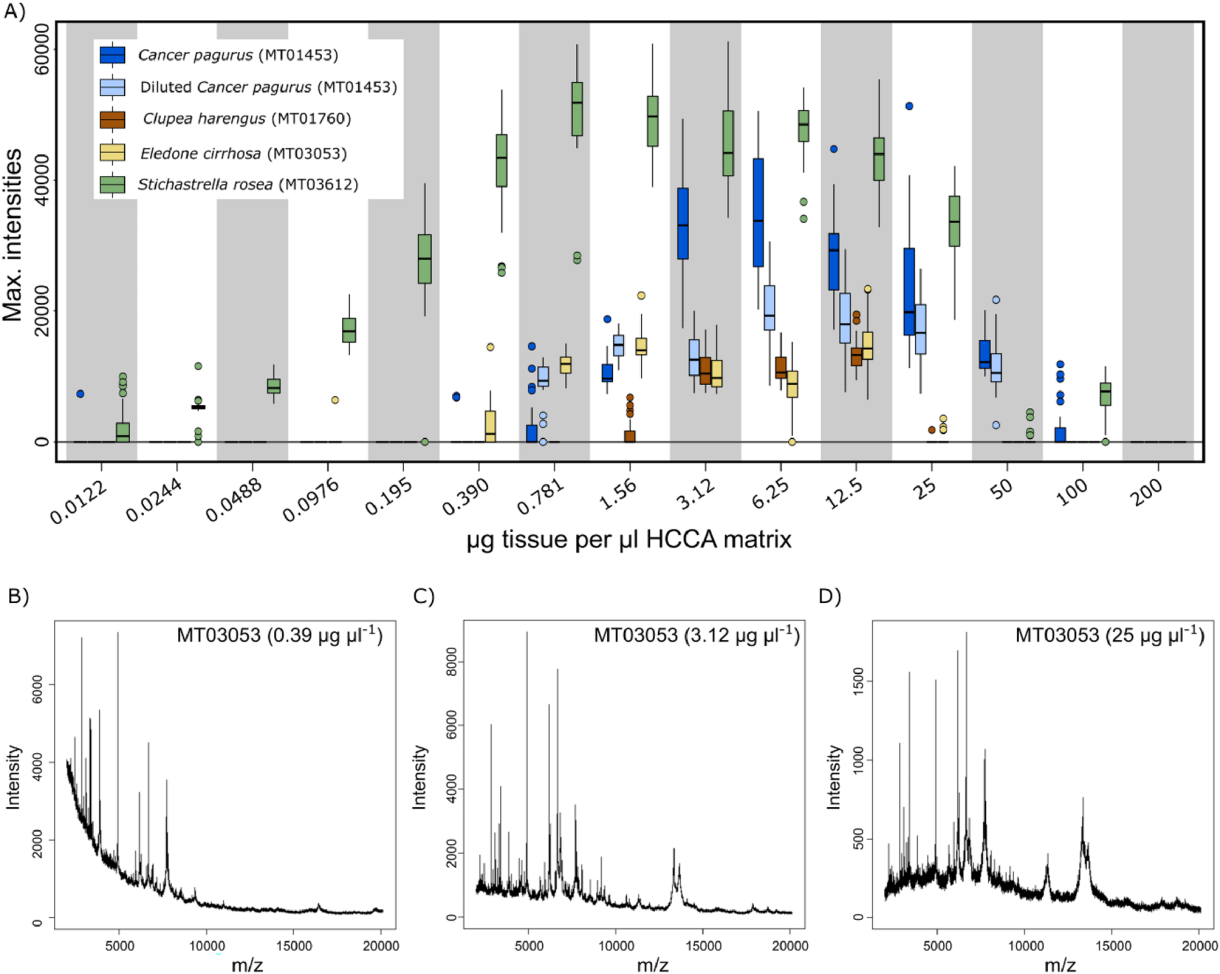
Results of the sample preparation test. All graphs show peak intensity on the y-axis. In (**A**), x-axis represents tissue:matrix ratio in µg per µl. In (**B–D**) m/z values (ratio of molecule mass and loading) are depicted on the x-axis. (**A**) Maximum intensities as a measure of quality for the different sample to HCCA matrix ratios assessed for four species. Additionally, for *Cancer pagurus* a dilution series (brown) was carried out. (**B**) Low quality spectrum at 0.39 µg µl^−1^ showing a high baseline at low molecule masses. (**C**) Good quality spectrum at a ratio of 3.12 µg tissue per µl matrix. (**D**) Low quality spectrum at 25 µg µl^−1^ showing stronger noise.

To determine the concentration range for successful measurements, weighted tissue samples were mixed with varying amounts of α-cyano-4-hydroxycinnamic acid (HCCA). In total, 15 different tissue/matrix concentrations were tested ranging from 0.01 to 200 µg µl^−1^ (Fig. 1A). Despite variations between samples, high quality mass spectra were generally assessed in a concentration range from to 3.1 to 12.5 µg µl^−1^. The largest concentration range for successful measurements was recorded for the echinoderm *Stichastrella rosea* (sample MT03612), with successful measurements across almost the entire concentration range. No measurements were obtained for concentrations of 200 µg µl^−1^. It was only when concentrations reached 12.5 µg µl^−1^ or lower that results were obtained for all specimens.

Good measurements were obtained from small tissue samples, when these were completely submersed in the HCCA solution within a 1.5 ml microcentrifuge tube (Fig. 1C). Before samples/matrix ratio was too low to detect a signal, an increase in baseline height in the lower masses was recorded (Fig. 1B). When sample to matrix ratio was increased, an increase in noise was observed (Fig. 1D). Quality improvement of spectra from high tissue-to-HCCA matrix ratios was achieved by dilution. This was tested using tissue from the crustacean *Cancer pagurus* (sample MT01453). Concentrations were diluted from an initial concentration of 200 µg µl^−1^ that resulted in no mass spectra at all. The measurements from diluted preparations then showed similar results as measurements made with the respective concentrations from undiluted sample preparations (compare Fig. 1A brown and red results).

### Optimize random forest (RF) model for classification

For application of RF as a method for classification, we evaluated how strongly the number of specimens per species influences model error. A repeated (n = 100) random sampling of two to eleven specimens for species with at least 11 specimens in the data set (n = 20) was carried out. This data was then used to create RF models and the out of bag error (OOB) was assessed as a quality criterion. Increasing the number of specimens per species resulted in a decrease of OOB error (Supplementary Fig. 1). With only two specimens per species the OOB error ranges from 0 to 0.375 with a mean error of 0.18 (SD = 0.073). With eleven specimens per species, the error ranges from 0.005 to 0.036 with a mean error of 0.019 (SD = 0.008). The decrease in OOB error goes nearly into saturation for n > 10. For further analyses, we chose n = 6 because the results show a strong decrease in OOB-error variability and a strong decrease in maximum OOB error at this point.

### Standardization of data processing

Different steps throughout data processing can have a severe impact on classification results. The effect of changing the different data processing steps was evaluated using the RF OOB error as an indicator. For each data set a RF model was trained and the OOB error recorded (Supplementary Fig. 2). Whereas alteration of baseline subtraction iterations generally only had little impact on RF OOB error, changing half window size (HWS) and the signal-to-noise ratio (SNR) for peak picking had greater effects (Supplementary Fig. 1). The generalized additive model (GAM) applied to find the most influencing factor shows that the OOB error is significantly influenced by alteration of the HWS (Table 1, p-value: 0.007) and SNR (Table 1, p-value: 0.001). A combination of 22 baseline estimation iterations, HWS of 7 and SNR of 3 resulted in the lowest OOB error of 0.032. These settings were used for further analyses.

**Table 1:** Results of the GAM analyses to detect the most important variable for data processing optimization.

	Estimate	Std.error	z value	P-value	Significance
Intercept	− 2.49	0.03	− 72.72	2e^−16^	***

	edf	Ref.df	chi.sq	P-value	Significance
Baseline iterations	1	1.001	0.125	0.72	
Peak detection HWS	1	1.00	7.39	0.007	**
SNR	1	1.00	10.2	0.001	*
	R-sq. (adj) = 0.686		Deviance explained = 68.7%		

Asterisks indicate levels of significance. * indicates P ≤ 0.05; ** indicates P ≤ 0.01; *** indicates P ≤ 0.001.

### Classification success

Finally, we tested the identification success based on MALDI-TOF MS data for each specimen in the data set by excluding the respective specimen and using the remaining reference data to identify it.

**Figure 2:**
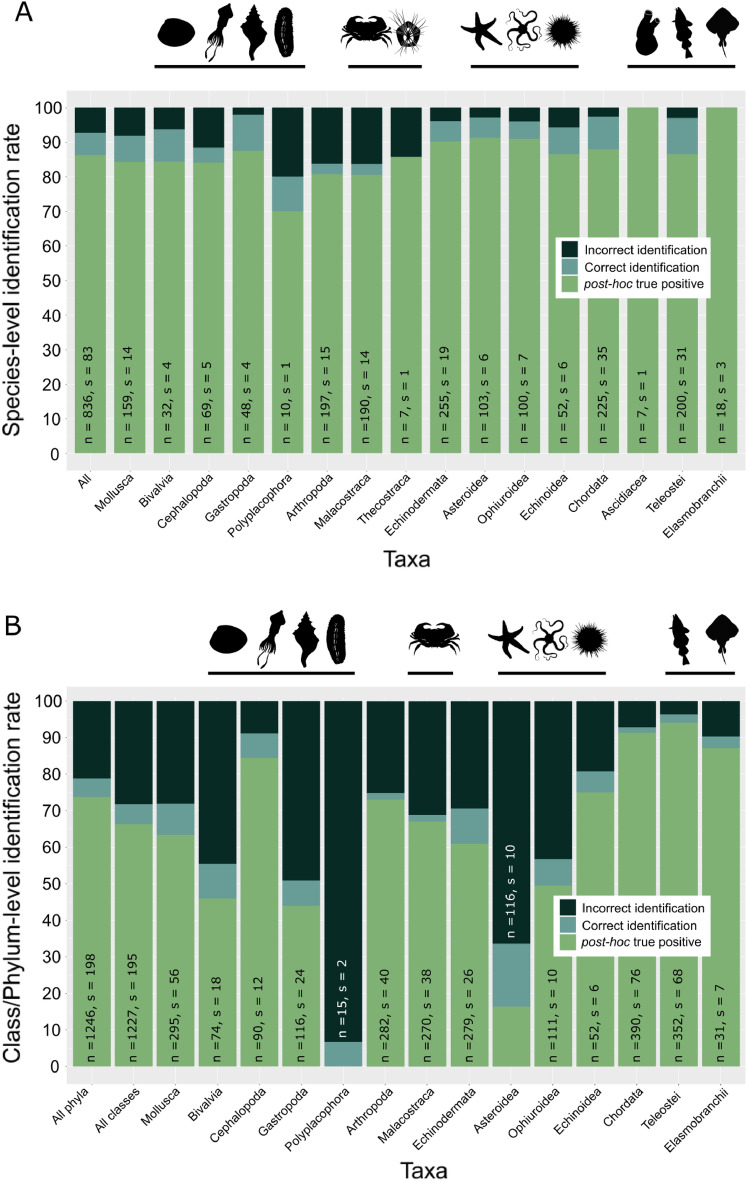
(**A**) Identification rate on species level displayed for all included phyla and classes separately. (**B**) Results of RF specimen identification to phylum and class level. Bars are divided into three categories relevant to the identification success. The darkest color displays the fraction of incorrect identifications, the intermediate color displays correct random forest identifications and the light color represents the percentage of specimens recognized as correct identifications by the *post-hoc* test.

Overall, 93% of the specimens (n = 775) were identified correctly and 86% (n = 721) were accepted as correctly classified by the *post-hoc* test (Fig. 2A). Identification for specimens of the classes Ascidacea, Teleostei, Elasmobranchii, Echinoidea, Ophiuroidea, Asteroidea, Bivalvia and Gastropoda resulted in success rates of more than 90%. For classes Cephalopoda and Thecostraca the identification success was still above 85%. Success rates lower than 80% were not recorded. Of the 61 misclassified specimens, 15 were assigned to the false species and recorded as correct identifications by the *post-hoc* test. Of all misclassified specimens, two were assigned to congeneric classes and rated as true positives by the *post-hoc* test, meaning these would have been misclassified and remain unrecognized.

### Case study: cryptic species

In the present data, the identification of the starfish *Astropecten irregularis* (Pennant, 1777) specimens from the North Sea serves as an example for closely related species that are still distinguishable by proteomic fingerprinting. In a previous study, this morphotype was found to consist of two major genetic clades with inter-clade distances in COI of up to 12%. Morphological differences were not determined so far. Both groups show different distribution patterns with overlaps^30^. Our data included specimens of both clades, *A. irregularis* 1 (n = 8) and *A. irregularis* 2 (n = 27).

**Figure 3:**
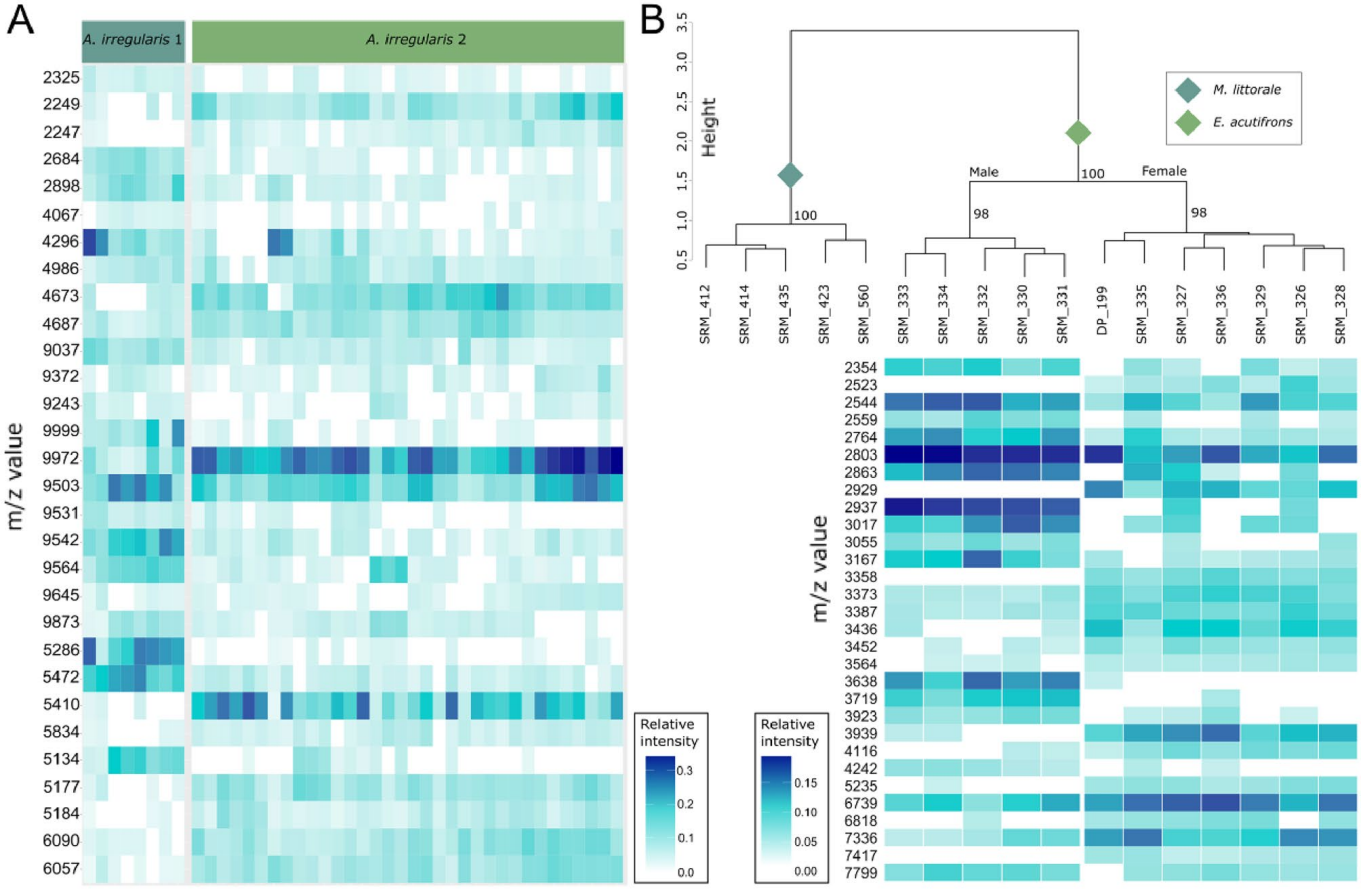
(**A**) The 30 most important peaks for differentiation of the starfish *A. irregularis* groups within the random forest model. Species according to COI delimitation are given on top. Molecule masses sorted by size are given on the left hand side. (**B**) Hierarchical clustering depicts differentiation of the copepod *E. acutifrons* specimens on sex level. Nodal bootstrap support is displayed at the nodes of the tree. The heatmap below the clustering results depicts the 30 most important mass peaks for sex-differentiation using a random forest model with color-coded peak intensities. Data from the marine copepod *Microarthridion littorale* (Poppe, 1881) from the same study was used here as an outgroup species. Relative intensities are color coded. Both images clearly show differences displayed in peak presence/absence and intensities between investigated groups on species level as well when looking hat different sexes of the same species.

Data processing settings were optimized for the sub-set of data (HWS = 9 and SNR = 8). Within a RF model produced from the data, a clear distinction between the two genetic groups was possible. None of the specimens was misassigned to the respective other group. This RF model was also used to find the most important variables for differentiation of the two groups using the Gini index, which shows the degree of dissimilarity of the respective variables^31^. The 30 most important variables are given in Fig. 3A. Whereas all peaks can be found in specimens of both groups, the intensities differ strongly allowing a clear differentiation of *A. irregularis* clades using proteome fingerprinting.

### Case study: sex determination

In previous research it was shown that sex determination may be possible in some species by analyzing the proteomic fingerprint^13^, however the data was not analyzed any further therein. In depth analyses emphasize these findings and show sex-specific protein patterns in the crustacean copepod *Euterpina acutifrons* (Fig. 3B). Mass peaks such as m/z 2523, 2929 and 7417 are female specific and not found in any of the male specimens. Others however, predominantly occur in male specimens (m/z 3638, 3719). Further mass peaks are evenly observed in measurements from both sexes but show intensity-pattern differences.

### Phyla and class models for identification

If a species is not part of a reference library, it may be desirable to obtain a higher level classification. To test if this is possible based on MALDI-TOF mass spectra of metazoans, species were systematically taken out of the RF training data set and classified with a RF model that was trained on higher taxonomic level but does not include any information on the respective species to be classified. Regarding all phyla together, a classification success of 81% (77% true positive rate (tpr)) was achieved with phyla-wise success rates ranging from 73% (64% tpr) in Echinodermata to 95% (92% tpr) in Chordata (Fig. 2B). On class level the combined success rate was 72% (66% tpr) ranging from 7% (0% tpr) in Polyplacophora, for which only two species were included in the data set, to 96% (94% tpr) in Teleostei.

For 31 taxa (n = 324), a congeneric species was included. Thus, it was tested if species have a higher affinity to be classified as a congeneric species in case the respective species is removed from the training data. Of these 31 taxa, 30% of specimens were classified as a congeneric species.

## Discussion

The aims of this study were (1) to evaluate the wide applicability of proteomic fingerprinting for species identification in marine science across different metazoan phyla and classes, (2) to identify critical steps in sample preparation and data processing, and (3) to contribute to the development of standard procedures and best practices for MALDI-TOF MS based metazoan classification in rapid biodiversity assessments. The general applicability to metazoans has been proven before^8,9,13,32–36^. However, here we show for the first time the applicability of this method to a large taxonomic range using a comprehensive data set with an overall species identification success rate of 93%.

Similar high identification success rates on species level were observed for individual metazoan groups^20,27,36–39^. Additionally, our results show that specimens absent from the reference library will be assigned to the correct phyla or class with a high probability implying some kind of phylogenetic signal on higher taxonomic level as was already reported for congeneric *Drosophila* before^40^. Testing if species would be classified as a congeneric species in the absence of the actual species was less promising in our study with only 30% of specimens being assigned to a congeneric species. This complies with other studies that only show occasional similarity of congeneric species e.g. in cluster analyses but without consistency across all congeneric species^11,13,26^.

In closely related species, morphological identification can often be complicated. Using proteomic fingerprinting, these problems can however be resolved as indicated by the analysis of the *A. irregularis* complex. Even though mass spectra show high similarities, distinct patterns of peak presence and absence as well as pronounced differences in relative peak intensities serve as good markers for species identification. Beyond mere species identification, the example of *E. acutifrons* shows the power of the method to differentiate specimens even on a sex level. This has been shown before for e.g. the fish species *Alburnus alburnus* (Linnaeus, 1758)^35^. Whereas authors focused on presence and absence of peaks, we were able to show that also relative intensities of certain mass peaks play an important role in differentiation of sexes. Prior studies on larger planktonic copepods have also shown a great potential for differentiation of developmental stages based on a proteomic fingerprint^17^.

Finally, we have shown the necessity of comprehensive reference libraries. Low numbers of specimens per species in reference libraries fail to provide sufficient information on species specific mass spectra features and intraspecific variability. Only with around nine to ten reference specimens per species, the identification error stabilizes on a constantly low level. This supports findings by Rakotonirina et al.^27^ who found an increase of identification score with increasing numbers of available main spectrum patterns. In general we would recommend to use more than three specimens per species and preferably to include around ten specimens for every species in a reference library.

MALDI-TOF MS can be used as a universal method for species identification of metazoan species. Due to the short preparation time, low costs^3,41^ and high identification success it can be a valuable tool in biodiversity assessments replacing time-intense morphological identification or costly DNA barcoding. Especially in cases of closely related or very similar species it can foster a rapid identification. The applicability of proteome fingerprinting for the differentiation of cryptic species was already shown and even in cases of morphologically very similar species, still differences were found^19,42^.

Tissue samples used in this work were obtained from specimens stored between seven to 12 years under partly unknown storage conditions. We assume working with fresh or recently fixed material would have resulted in even higher identification success rates. This is supported by the high mass spectra quality obtained from fish species, which were extracted and put into freezer storage almost immediately after sampling (personal communication Knebelsberger). The adverse effect of fixation and storage on resulting mass spectra quality in metazoans was investigated several times and supports this assumption^27,43^. We received good results for storage at − 20 °C and also for long-term storage at − 80 °C, thus we recommend cold storage of samples at − 20 °C, until further systematic analyses will specify threshold temperatures for short- (months) or long-term (years) storage.

Our tests have shown that sample concentration is pivotal to obtain good quality mass spectra. While too low sample/matrix ratios will result in lower intensities and a higher baseline, too much tissue will increase the noise in the data and result in unsuccessful measurements. For all investigated taxa, the same sample preparation method was used; however attention must be paid to the correct ratio of matrix and compound to be analyzed. This allows the wide application of this method without adaptation of the protocol to a certain species as it would be necessary for methods such as COI barcoding where certain groups would need highly specific sets of amplification primers^44,45^ and adjustment of PCR settings. We expect that mass spectra quality could be further improved with more elaborate preparation protocols. This would however counteract the advantage of this method being rapid, user-friendly and straightforward compared to other methods such as COI-barcoding. A critical aspect for the future establishment of this method is also the development of objective evaluation criteria for the sufficient quality of a spectrum for species identification and the procedures to analyze it. Such evaluation methods will be necessary to ultimately facilitate the integration of numerous species spectra into cross-laboratory databases.

Much effort is put into optimizing mass spectra quality by adjusting different preparation protocols^46,47^ or developing methods for steps such as baseline correction, smoothing or peak picking^48,49^. Methods are adjusted either to increase classification success or to obtain better mass spectra reproducibility. Here, we tested the influence of certain steps during data processing on classification success focusing on the important steps for peak detection. Whereas baseline subtraction and adjustment of a SNR value both aim at reducing noise within the data, adjusting the HWS influences the peak picking resolution. Thus, by decreasing the HWS during peak detection, the number of peaks will increase as the highest peak within the HWS will be the detected. This will result in peaks of very similar size being recognized as distinct peaks, rather than being put together in a single bin. This does also explain the high effect of both parameters SNR and HWS compared to baseline subtraction. Baseline subtraction is constrained towards reducing instrument-dependent noise. Adjustment of the SNR value will however, like HWS alteration, affect the number of more dominant peaks and thus the general resolution of the mass spectra. Hence, more species-specific information is retained and more information is available for classification. Based on our results, rather than testing all variables, adjusting SNR and HWS should be adequate to optimize the data pipeline. However, it needs to be emphasized that this pipeline aims at optimizing species identification and may not be adequate for investigation of intraspecific variability as was shown elsewhere^16^.

**Figure 4:**
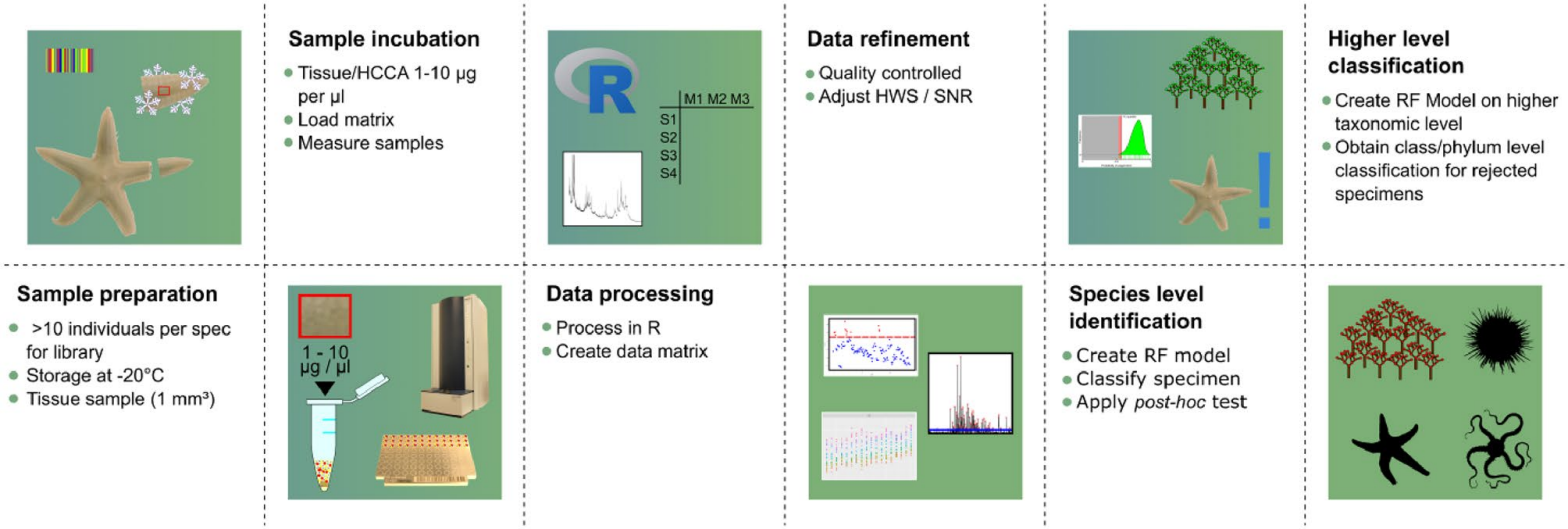
Proposed workflow from specimen to classification. Tissue samples should be stored (especially for long term) at cold temperatures until further processing. From our tests, we suggest sampling ten specimens per species. A small subsample (1–10 µg tissue per µl matrix) is incubated in HCCA Matrix and the solution is transferred to a target plate for measurement. Results are imported to R and data are quality controlled and critical parameters of the model for classification (SNR/HWS) are adapted. Classification is done using the optimized RF model. The *post-hoc* test from the R package RF Tools is applied to verify classification. If classification is rejected, phylum/class/family level models may result in a higher taxonomic classification. The R logo is © 2016 The R Foundation and is used under the CC-BY-SA 4.0 license.

In summary, we propose a workflow applicable for any metazoan species or tissue sample to be identified: A comprehensive reference library is needed with species level identification by morphological or molecular approaches (Fig. 4). In the lab, a small tissue (up to 1 mm^3^) is retrieved and incubated for at least 5 min in the HCCA-matrix solution. Of the resulting extract, 1 to 1.5 µl are transferred to a target plate for measurement. Data processing is carried out in R (Fig. 4). Mass spectra quality is done by eye and supported by R-packages such as MALDIrppa^50^. Finally, based on previously assessed species identification, data processing can be optimized to obtain ideal settings for classification. Depending on our results this can be narrowed to adjustment of HWS- and SNR-value. Based on the reference library, a RF model can be calculated for specimen identification (Fig. 4). Applying a *post-hoc* test will provide further support for the identification. If classification is not well supported, a RF model on class or phyla level can be applied to obtain higher-level classification.

## Conclusion

MALDI-TOF MS was proven an easy to apply, cost-effective and time-saving tool for identification across taxa. It is especially feasible in applications where mere species identification is desired, for example in biodiversity assessments By the standardized workflow based on a wide range of marine metazoan specimens can be identified quantitatively and effectively on species level thereby bypassing some of the high requirements associated with genetic methods, such as access to special laboratories, searching for primers etc. We want to highlight here that proteomic fingerprinting will be due to its simplicity, reliability and efficiency a valuable supplement to the molecular toolbox for taxonomy.

## Methods

### Sample material

Tissue for measurements was taken from the marine organisms tissue bank of the Senckenberg am Meer, German Centre for Marine Biodiversity Research, which was established using samples from numerous studies^30,51–57^ (Supplementary Table S1 for accession numbers) on North Sea metazoans. The material from this collection was taken from specimens processed for COI-barcoding to create reference libraries for a variety of marine animal groups. During this process, tissue samples of the respective specimens were stored in ethanol at − 80 °C. Tissue samples were available for Bivalvia (muscle, 18 species), Cephalopoda (muscle from arm, 12 species), Gastropoda (muscle from foot, 24 species), Polyplacophora (muscle from foot, 2 species), Ascidiacea (tissue, 1 species), Teleostei (muscle, 67 species), Elasmobranchii (muscle, 7 species), Malacostraca (muscle from foot or chelae, 39 species), Thecostraca (muscle from foot, 1 species), Pycnogonida (leg fragment, 1 species), Asteroidea (tube feet, 10 species), Ophiuroidea (tissue from arm, 10 species) and Echinoidea (tissue from the base of the tubercle, 6 species) (n_species_ = 198, n_specimens_ = 1246).

### Sample preparation

The basic protocol of sample preparation was the same for all analyzed tissue samples. A very small tissue fragment (< 1 mm^3^) was incubated for 5 min in HCCA as a saturated solution in 50% acetonitrile, 47.5% molecular grade water and 2.5% trifluoroacetic acid. Tissue from crustacean *Cancer pagurus* Linnaeus, 1758, the fish *Clupea harengus* Linnaeus, 1758, the cephalopod *Eledone cirrhosa* (Lamarck, 1798) and the echinoderm *Stichastrella rosea* (O.F. Müller, 1776) was used to find an optimal tissue to HCCA matrix ratio. Tissue was weighed on a METTLER TOLEDO XS3DU micro-balance and the amount of matrix was adjusted to tissue weight to obtain the desired ratios ranging from 0.012 to 200 µg µl^−1^. After incubation, 1.5 µl of the solution was transferred to 10 spots on a target plate, respectively. Mass spectra were measured with a Microflex LT/SH System (Bruker Daltonics) using method MBTAuto. Peak evaluation was carried out in a mass peak range between 2 and 10 k Dalton (Da) using a centroid peak detection algorithm, a signal to noise threshold of 2 and a minimum intensity threshold of 600. To create a sum spectrum, 160 satisfactory shots were summed up.

Resulting from observations during this initial test, a fast applicable protocol was developed without the need to weigh each tissue sample. Our tests allow us to identify inferior sample-to-matrix ratios and thus adapt the sample preparation. Also they showed that the spectrum quality is sufficient across a wide spectrum of tissue-to-matrix ratios. Thus, we concluded that the Matrix volume to be added to tissue samples can be adjusted depending on tissue volume, so that tissue samples are always completely covered by HCCA matrix with a small layer (ca. 1 mm) of supernatant. Samples were incubated for 5 min and 1.5 µl of the solution were transferred to a single spot on a target plate for measurement. Each spot was measured between two to three times.

### Mass spectra processing in R

Mass spectra data was imported to R^58^ using MALDIquantForeign^59^ and further processed using MALDIquant^60^. Mass spectra were trimmed to an identical length from 2 to 20 kDa. Subsequently, spectra were square root transformed, smoothed using Savitzky Golay method^61^, baseline corrected using SNIP approach^62^ and normalized using total ion current (TIC) method.

Spectra were quality controlled using the command ‘screenSpectra’ from the R-package MALDIrppa^50^. Mass spectra with a notably high a-score were checked by eye and discarded if mass spectra were of bad quality. If due to this, only a single specimen for a certain species was retained, the remaining specimen was discarded from the data set.

### Evaluation of random forest model for identification

Besides initial sample preparation and subsequent data processing, we tested how to improve a random forest (RF) model used for species identification. Optimal number of trees and variables was tested in a previous study^63^. Here we assessed the effect of minimum number of specimens per species category on the resulting model power. We sampled the dataset using two to 11 specimens per species including only species with at least 11 specimens per class (n = 20). For each minimum number of specimens, 100 data sets were sampled using ‘sample_n’ from R-package dplyr^64^, a RF model was created and the OOB errors assessed accordingly.

### Standardization of data processing

Based on literature research and own observations, three data processing steps were identified, which may have a severe impact on data and the resulting quality of a random Forest (RF) classification model^65^. (I) Iterations of baseline subtraction: this is a first manipulation step to reduce chemical noise and is carried out iteratively^66^. Increasing iterations will result in loss of low intensity peaks. (II) Signal to noise ratio (SNR) during peak picking: an increase in SNR will exclude signals of low intensity. The higher the SNR value, the less peaks will be kept. (III) Half window size (HWS) during peak picking: within the HWS the peak with the highest intensity will be chosen as the resulting peak during peak picking. The higher the HWS is chosen, the less peaks will be picked across an entire mass spectrum range.

Interactive effects of these data processing steps were tested using the classification success by a random forest model as target variable: iterations of baseline estimation and peak detection HWS were varied both between 5 to 30 and SNR from 3 to 20. In total, 12,186 analyses were carried out. In all cases, peak binning using ‘binPeaks’ from R-package MaldiQuant was repeated until the number of variables in the data did not further change. The RF model (ntree = 2000 and mtry = 35) was trained on the Hellinger transformed peak intensities as suggested by Rossel and Martínez Arbizu^43^. The RF out-of-box (OOB) error was used as measure for classification success. For these analyses, based on the results from RF-model evaluation, only species were included with at least six specimens. To investigate the main drivers of classification success, a generalized additive model (GAM, family: binomial; link function: logit) was calculated.

### Testing the classification success

In concordance with the results from the previous tests, only species with at least six specimens were included in the model. Mass spectra from these species were processed according to the results from the test on variation of HWS (7), SNR (3) and baseline iteration (22). To test the overall classification success on species level, single specimens were separated from the RF training data set and subsequently identified using this model. After classification, the *post-hoc* test by Rossel and Martínez Arbizu^63,66^ using the R-package RFtools (https://github.com/pmartinezarbizu/RFtools) was applied to verify RF classification. This *post-hoc* test uses the empirical distribution of RF assignment probabilities from the RF model and compares the assignment probabilities of newly classified specimens to this distribution. Whereas classified specimens with assignment probabilities falling within this empirical distribution are considered true positive (tp), specimens with probabilities of assignment significantly different to this distribution considered false positive (fp).

### Case studies

In order to show the applicability of MALDI-TOF MS, we present two model cases. First, data of the North Sea starfish *Astropecten irregularis* (Pennant, 1777) were investigated based on MALDI-TOF mass spectra. This species was found to be genetically divergent^30^ while revealing a high morphological similarity. Differentiation of species was tested using RF models. Furthermore, data on the crustacean *Euterpina acutifrons* (Dana, 1847) from Rossel and Martínez Arbizu, 2019 was analyzed to show the applicability for sex level differentiation using hierarchical clustering and RF. Based on the Gini index the 30 most important peaks for species/sex differentiation in a RF model were extracted and investigated to show the expression within the respective groups.

### Phyla and class models for identification

To test whether specimen can be identified on an above-species level, a RF model containing only class- and phylum-categories was applied. All spectra from species to be classified were excluded from the model to evaluate its use for specimen not included in a library. The respective specimens were identified using the model and the predicted class/phylum was tested with the RF *post-hoc* test. To test classification on phylum level, 1246 specimens from 198 species were included. On class level, 1227 specimens from 195 species were analyzed.

### Supplementary Information

Supplementary Legends.

Supplementary Figure 1.

Supplementary Figure 2.

Supplementary Table S1.

## Data Availability

All mass spectra is available at Data Dryad (10.5061/dryad.7pvmcvdzf). Relevant R-Scripts are stored alongside the raw data.
