# Supplementary material for: A universal tool for marine metazoan species identification: towards best practices in proteomic fingerprinting
Source: Sci Rep. 2024 Jan 13;14:1280. doi: 10.1038/s41598-024-51235-z (PMC10787734; doi:10.1038/s41598-024-51235-z)
Supplement: Supplementary file 1 — Supplementary Legends. [file 41598_2024_51235_MOESM1_ESM.docx]

Supplementary Fig. 1: Random forest model quality assessed with increasing number of specimens per species. For each number of specimens, 100 data sets were created by random sampling. The OOB error (y-axis) decreases with increasing number of specimens (x-axis) and starts going into saturation. Thus, around 10 specimens per species are generally recommended to obtain a high quality model.

Supplementary Fig. 2: Out of box error of the random forest model (OOB error on y-axis) for the different combinations of baseline subtraction iterations, signal to noise ratio (SNR) and half window size (HWS) during peak picking. Each box represents the number of baseline iteration steps ranging from 5 to 30. The x-axis displays the SNR value ranging from 3 to 20. Colors indicate different HWS ranging from 5 to 30. The results are shown for all 12,186 variable combinations.
