# Supplementary figures and images for: A universal tool for marine metazoan species identification: towards best practices in proteomic fingerprinting
Source: Sci Rep. 2024 Jan 13;14:1280. doi: 10.1038/s41598-024-51235-z (PMC10787734; doi:10.1038/s41598-024-51235-z)

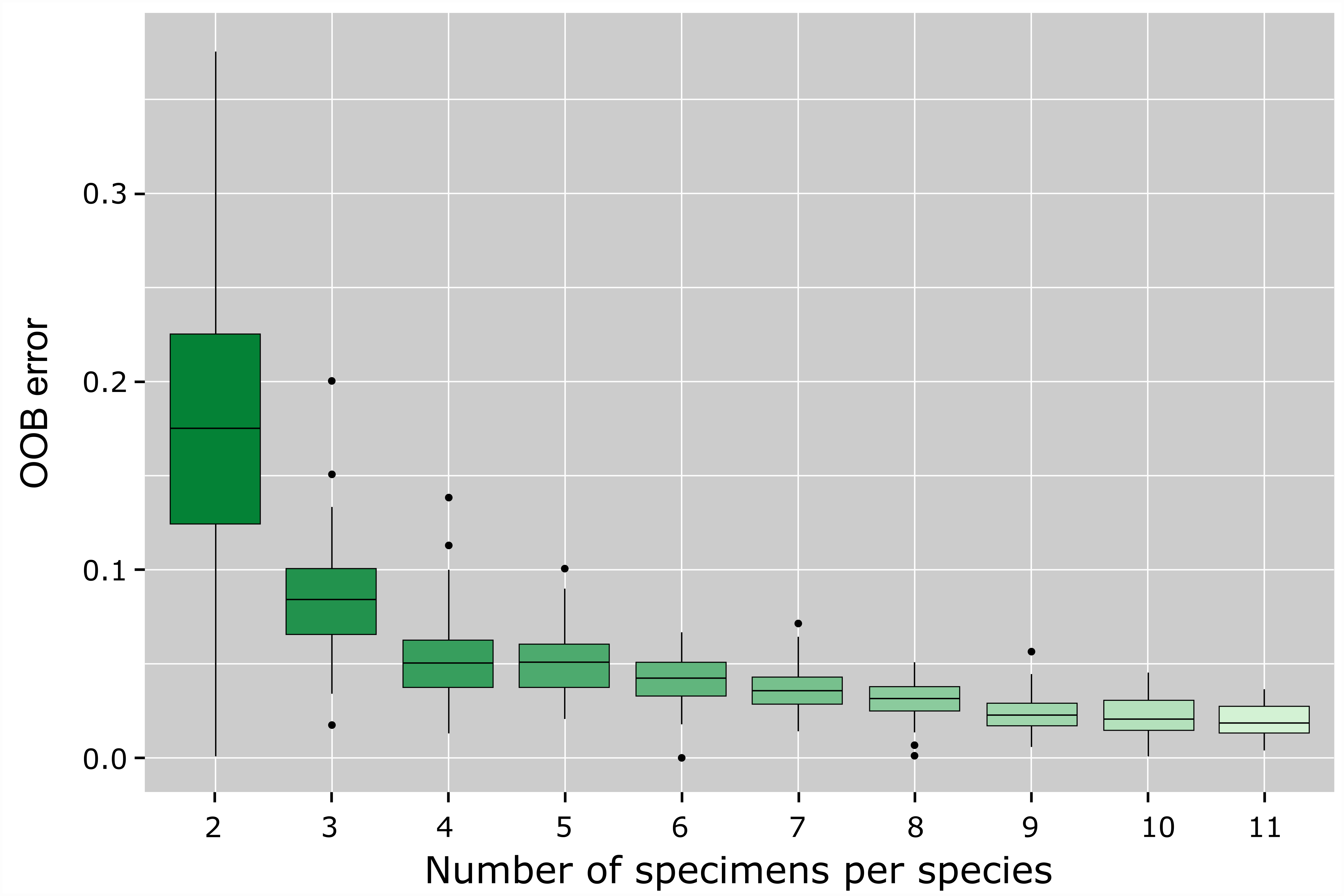

Supplement: Supplementary file 3 — Supplementary Figure 2. [file 41598_2024_51235_MOESM3_ESM.png]
